# Immobilization of soybean peroxidase enzyme on hierarchical zeolite-ordered mesoporous carbon nanocomposite and its activity†

**DOI:** 10.1039/d4ra07503j

**Published:** 2025-02-20

**Authors:** Prabhu Azhagapillai, Karthikeyan Gopalsamy, Israa Othman, Nada I. Alhatti, Mohammad Abu Haija, Syed Salman Ashraf

**Affiliations:** a Department of Chemistry, Khalifa University P. O. Box 127788 Abu Dhabi United Arab Emirates prabhu.au@gmail.com; b Department of Biological Sciences, Khalifa University P. O. Box 127788 Abu Dhabi United Arab Emirates syed.ashraf@ku.ac.ae; c Department of Chemistry and Biotechnology, Baikal School of BRICS, Irkutsk National Research Technical University 83, Lermontov Street 664074 Irkutsk Russian Federation; d Center for Catalysis and Separations (CeCaS), Khalifa University P. O. Box 127788 Abu Dhabi United Arab Emirates; e Advanced Materials Chemistry Center (AMCC), Khalifa University P. O. Box 127788 Abu Dhabi United Arab Emirates; f Center for Biotechnology (BTC), Khalifa University P. O. Box 127788 Abu Dhabi United Arab Emirates

## Abstract

Immobilization of enzymes on inorganic supports such as silica and carbon materials is an effective approach for chemical surface modification. In this work, hierarchical zeolite (HZ-SAPO's) materials were fabricated by a modified method, and mesoporous carbon (CMK-3) was synthesized using the SBA-15 mesoporous silica as a template. A variety of biocatalysts was prepared using HZ-SAPO with CMK to furnish the nanocomposite biocatalyst. The functionalization of amine group with APTES was done which was further immobilized by Soybean Peroxidase (SBP) enzyme. The material was subjected to a comprehensive characterization process utilizing numerous systematic methods, including X-ray diffraction, N_2_ adsorption–desorption isotherms, Raman spectroscopy, scanning electron microscopy, high-resolution transmittance electron microscopy, and attenuated total reflectance Fourier transform infrared spectroscopy. The pH effect on the immobilized enzyme was examined and compared to that of SBP. Further, the assessment of repeated usability of immobilized SBP with successive cycles was carried out.

## Abstract 2

Immobilization of enzymes on inorganic supports such as silica and carbon materials is an effective approach for chemical surface modification.

## Graphical abstract

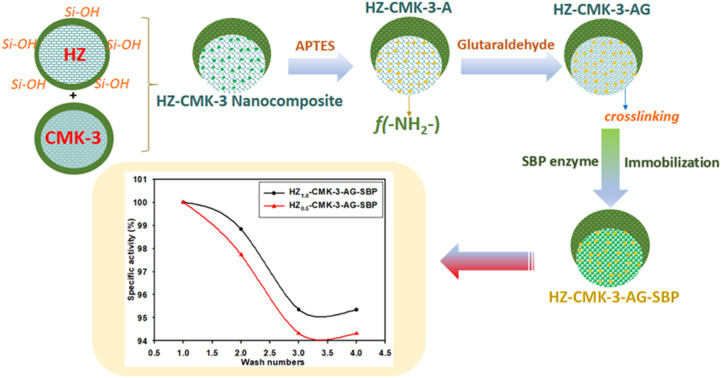

## 1. Introduction

Enzyme-based biocatalytic processes have become a crucial task for the present-day industrial sectors.^1,2^ Biocatalytic materials involve the employment of enzymes and the modification of enzyme structures to attain stability, activity, and specificity which determine their optimal use in various industrial processes. In this regard, the immobilization of enzymes with solid supports such as silica, carbon, and inorganic metals is one effective approach to tune the bioactivity of the composite hybrids. Enzyme immobilization refers to the process of attaching enzymes to specific solid support, often to improve their stability, reuse, and specificity in biocatalytic reactions.^3^ Such an immobilized enzymatic system on inorganic materials is indispensable in a way that is protected and recovered in the potential application.^4^ For instance, modification of inorganic surfaces and the effects of immobilization could alter the material properties of the enzyme carriers.^5,6^ Furthermore, the biocatalytic features, practical regeneration, and enzyme leakage has been probed and still remain a challenge.^7^ More importantly, inorganic micro-nanomaterials offer viability in enzyme immobilization due to their unique physicochemical properties, such as tunable pore size, pore volume, large surface area, hydrothermal, mechanical, and mass-transfer resistance.^8,9^ Such hybrid materials have been explored as suitable biocatalysts in pollutant degradation, pharmaceutical removal, sensing, drug delivery, and the food-processing industry.^10–13^

Efficient solid supports involve zeolites, mesoporous silica, and mesoporous carbon where the inorganic bonding could be treated with targeted functional groups by functionalization and crosslinking agents.^14–17^ Among them, hierarchical zeolites are a class of microporous materials that have both macro- and mesopores, in addition to their microporous structure.^18,19^ The combination of pore sizes and structures makes them attractive for applications in areas such as catalysis, separations, and adsorption. In particular, mesopore surfaces and active sites in the hierarchical zeolites contribute in adsorption properties.^20^ Mesopore walls with silanol groups in hierarchically constructed meso-microporous zeolites can be functionalized with organic moieties for superior hydrothermal stability and reactive active sites.^21^ Such functionalized hierarchical support materials could be a choice for the immobilization of enzymes.

Other solid materials such as activated carbons are commonly explored for the immobilization of enzymes due to available binding sites for enzyme bonding.^22–24^ Owing to the restricted pore size, disordered structure, and inert functionalities in activated carbon, their application is limited in functionalization, crosslinking as well as immobilization. In this context, an inorganic support material with a comprehensive pore structure and far greater surface area is necessary to benefit easy diffusion of the substrate molecules thereby achieving a higher reaction rate.^25^ In this regard, mesoporous carbon materials are another promising solid support with a large surface area, highly ordered mesopores, and possess higher hydrothermal resistance.^26,27^ CMK-3, a mesoporous carbon with an ordered pore structure that has been produced utilizing the hard template method from silica SBA-15, has several advantages over activated carbon for enzyme immobilization. Firstly, CMK-3 has a higher surface area and a larger number of binding sites compared to activated carbon, making it more suitable for immobilization.^28^ Secondly, the mesoporous structure of CMK-3 enables it to have a well-defined pore size and distribution, which can improve the stability of the enzyme and enhance its activity.^28,29^ Additionally, the uniform pore size and the ordered structure of mesoporous carbons have control over the adsorption and large immobilization capacity.^30^

Mainly, the composed advantages of hybrid hierarchical zeolites and mesoporous carbon characteristics is beneficial in effective functionalization and enzyme immobilization offering potential hybrid biocatalysts. The most favored step by step functionalization followed by immobilization of enzyme with surface properties are vital. Such immobilization has been successfully carried out to synthesize sustainable biocatalysts with exceptional operational stability and reusability.^31,32^ From the above state of the art, herein, a hierarchical zeolite-ordered mesoporous carbon (HZ-CMK-3) nanocomposite material has been fabricated step-by-step by the process of synthesizing HZ-SAPOs and CMK-3 separately under optimized reaction conditions. Then the HZ-CMK-3 was functionalized with (3-aminopropyl) triethoxysilane (APTES) followed by glutaraldehyde (GA) crosslinking to obtain the surface modifications of the inorganic material. The surface-modified and functionalized HZ-CMK-3 gives more access for enzyme immobilization, where the SBP enzyme was successfully immobilized onto the HZ-CMK-3-APTES-GA hybrids. The prepared materials were characterized by various physico-chemical analytical techniques to elucidate their structure, morphology, texture, functional groups, and composition. The activity study and regenerability of the SBP immobilized biocatalyst were investigated to understand the extent of practical leaching and reusability. In this approach, the hierarchical zeolite component provides the nanocomposite, with a unique hierarchical structure, which includes both macro- and meso-scale pores, providing the material with a high surface area and pore volume. Such hierarchical assembly with interconnected wide pores reduces the diffusion problems in reaction kinetics and is also readily available for functionalization. On the other hand, CMK-3 with an ordered pore structure provides stability and robustness to the material, as well as additional surface area for enzyme immobilization. Furthermore, the HZ-CMK-3 nanocomposite is highly stable, even under harsh conditions, making it ideal for use in various industrial applications. Such hybrid functional materials are highly promising for enzyme immobilization and biocatalytic reactions, combining the benefits of hybrid structural, functional, and textural properties.

## 2. Materials and methods

Pluronic P123 (MW = 5800) [pluronic P123 is a triblock copolymer with the chemical structure (EO)20–(PO)70–(EO)20, where EO stands for ethylene oxide and PO stands for propylene oxide], tetraethyl orthosilicate (TEOS), butanol (C_4_H_9_OH), hydrochloric acid (HCl), sulfuric acid (H_2_SO_4_), hydrofluoric acid (HF), sucrose, triethylamine, pseudo-boehmite, phosphoric acid, colloidal silica, glutaraldehyde (GA), and (3-aminopropyl) triethoxysilane (APTES) were used as raw materials from the Sigma-Aldrich. The chemical materials were utilized directly without any additional purification. The SBP enzyme, which possesses a specific activity of 2700 IU mg^−1^ (1 mg mL^−1^, 26 micro M), was provided by Bio-Research Products situated in North Liberty, IA, USA. All experimental procedures were conducted using universal buffers containing 0.2 M potassium hydrogen phosphate (K_2_HPO_4_) and 0.1 M citric acid (C_6_H_8_O_7_).

### 2.1. Synthesis of mesoporous silica (SBA-15)

A solution of 4 grams of pluronic P123 was prepared by dissolving it in 30 mL of distilled water and stirring at room temperature for 4 hours. Subsequently, 120 grams of 2 M HCl was added to the solution, followed by stirring for an additional 2 hours at 40 °C. To this mixture, 9.6 grams of tetraethyl orthosilicate (TEOS), serving as the silicon source, was introduced, and the solution was stirred vigorously for 24 hours. The resulting gel was transferred to an autoclave and subjected to hydrothermal treatment at 100 °C for 48 hours. After cooling to room temperature, the product was filtered, thoroughly rinsed with deionized water, and dried overnight at 60 °C. Finally, the dried material was calcined by heating at a rate of 1 °C min^−1^ to 540 °C and maintained at this temperature for 6 hours.

### 2.2. Synthesis of hierarchical zeolite (SAPO)

The hierarchical SAPO intergrowth material was prepared using a hydrothermal synthesis method.^33^ Initially, a gel mixture was formulated with silica sol, pseudo-boehmite, H_3_PO_4_, and a single template, TEA (triethylamine). The molar composition of the gel was Al_2_O_3_ : 0.22SiO_2_ : 1.06P_2_O_5_ : 3TEA : 60H_2_O. The crystallization process was conducted in two stages: first at 140 °C for 2 hours, followed by 180 °C for 6 hours. The resulting catalyst powders were separated by centrifugation and thoroughly washed with deionized water, ethanol, and acetone to remove impurities. The solid products were dried at 80 °C for 12 hours and subsequently calcined in air at 550 °C for 5 hours to eliminate the template. The final product is referred to as HZ (SAPO).

### 2.3. Synthesis of mesoporous carbon (CMK-3)

Ordered mesoporous carbon (CMK-3) was synthesized using mesoporous silica (SBA-15) as a hard template and sucrose as the carbon precursor. The synthesis involved impregnating the SBA-15 structure with a sucrose solution. Initially, 1.0 g of SBA-15 was added to an aqueous solution containing 1.25 g of sucrose, 0.14 g of H_2_SO_4_, and 5.0 g of deionized water. The mixture was thermally treated at 100 °C for 6 hours, followed by heating at 160 °C for an additional 6 hours. This impregnation process was repeated using 0.8 g of sucrose, 0.09 g of H_2_SO_4_, and 5.0 g of deionized water, followed by the same thermal treatment. The impregnated silica template was then carbonized at 900 °C for 6 hours under a nitrogen atmosphere. To remove the silica template, the carbonized material was treated with HF solution, followed by filtration, thorough washing with water and ethanol, and drying overnight at 80 °C, yielding the final CMK-3 product.

### 2.4. Synthesis of HZ (SAPO)-CMK-3 nanocomposites

To evaluate the materials performance in synthesizing the nanocomposite, two different ratios were optimized. In the first synthesis, 0.5 g of HZ (SAPO) and 0.5 g of CMK-3 were mixed with 10 mL of 1 M NaOH in a beaker and stirred for 12 hours at room temperature. The temperature was then gradually increased to 80 °C, and stirring continued for an additional 8 hours. The resulting product was thoroughly washed with distilled water until the pH of the wash water reached 7. The solid black powder was dried overnight in an oven at 80 °C, yielding the final HZ-CMK-3 nanocomposite, denoted as HZ_1.0_-CMK-3.

A second nanocomposite with a different ratio was synthesized using 0.25 g of HZ (SAPO) and 0.5 g of CMK-3, following the same procedure. This product was designated as HZ_0.5_-CMK-3.

### 2.5. Functionalization of APTES over HZ_*x*_-CMK-3

To functionalize the material, 0.5 g of HZ_*x*_-CMK (each) was placed in a 100 mL round-bottom flask with 50 mL of toluene and stirred for 15 minutes. The mixture was then subjected to ultrasonication for 15 minutes. Following this, 1 mL of APTES was added to the solution, and stirring was continued at 80 °C for 24 hours. After the reaction period, the mixture was filtered and washed sequentially with deionized water, ethanol, and acetone to remove any impurities. The resulting blackish powder was dried at 80 °C for 12 hours, yielding the final product, designated as HZ_*x*_-CMK-3-A.

### 2.6. Crosslinking of glutaraldehyde over HZ_*x*_-CMK-3-A

To enhance the materials activity, a crosslinking process with glutaraldehyde (GA) was carried out. For this, 150 mg of HZ_*x*_-CMK-A material was suspended in 15 mL of a 1 wt% GA solution and stirred for 4 hours. The resulting solid was then filtered and thoroughly rinsed with 300 mL of deionized water to remove any excess reagent. Finally, the product was dried overnight at 40 °C under vacuum, yielding the final material, designated as HZ_*x*_-CMK-3-AG.

### 2.7. Immobilization of soybean peroxidase over HZ_*x*_-CMK-3-AG

**Scheme 1:**
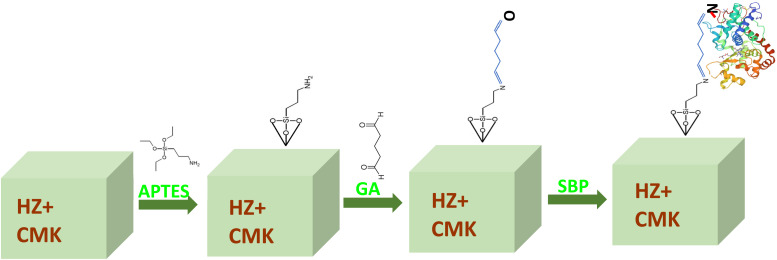
Schematic representation of the synthesis of functionalized HZ-CMK-3 nanocomposite materials and immobilization of SBP.

A total of 100 mg of soybean peroxidase powder (extracted from soybean seeds) was mixed with 10 mL of buffer solution and gently shaken for 15 minutes. The mixture was then stored overnight at 4 °C in a refrigerator.^34^ The solution was subsequently stirred gently and centrifuged to separate the bottom colloidal components. The supernatant (top layer) was filtered using filter paper and stored at 4 °C for further examination. A schematic representation of the synthesis of HZ-CMK-3 nanocomposite materials, along with their functionalization, crosslinking, and SBP enzyme immobilization, is shown in Scheme 1.

### 2.8. Activity assay

The spectrophotometric assay was employed to measure the enzymatic activity of both free and immobilized soybean peroxidase. ABTS (2,2′-azino-bis(3-ethylbenzothiazoline-6-sulfonic acid) was used as the substrate, and the measurements were conducted at room temperature. The microplate reader was used to monitor the absorbance changes specifically at 420 nm (acculab, MIR-700, Canada).

### 2.9. Characterization

The structure and crystallinity features were analyzed by high angle powder X-ray diffraction (XRD) characterization performed on a Bruker D2 Phaser X-ray diffractometer by using Cu Kα radiation (*λ* = 1.5418 Å). The small angle X-ray diffraction (XRD) patterns of the mesoporous materials were analyzed by the Rigaku diffractometer using Cu Ka radiation of wavelength 0.154 Å. The diffractograms were recorded in the 2-theta range 0.4–5 with a step size of 0.01 and a step time of 10 s.

The BET surface area of the samples was determined using the multipoint nitrogen adsorption–desorption method at a liquid nitrogen temperature (−196 °C) with an ASAP 2000 (Micromeritics) instrument. Before the nitrogen sorption experiment, the samples were outgassed at 623 K for 6 hours to remove moisture.

JEOL JEM-4000EX equipment operated at 400 kV with a field emission gun was used to record transmission electron microscopic (TEM) images from the thin edges of particles supported on a porous carbon grid.

Bruker ALPHA-platinum ATR was utilized to conduct Fourier transform infrared (FTIR) spectroscopy measurements in the range of 400–4000 cm^−1^ for 32 scans.

Raman spectroscopy was employed to determine the Raman bands of the hierarchical nanocomposite materials. The spectra were collected using a Horiba LabRamanHR microscope equipped with a Coherent Sapphire laser operating at a wavelength of 630 nm (blue laser). A thermoelectrically cooled CCD detector with a 1800 lines per mm grating was used to disperse the signal. The excitation beam was focused on the sample using a 50× objective lens. Raman spectra were recorded in the 1200–100 cm^−1^ region after 50 scans at a resolution of 2 cm^−1^.

Scanning electron microscopy (SEM) was used to obtain the surface morphology of the mesoporous carbon, and hierarchical nanocomposite samples. The SEM used for this purpose was the Quanta 3D, operated at 30 kV. Prior to the SEM analysis, the samples were coated with a thin layer of gold.

## 3. Results and discussion

### 3.1. XRD

**Fig. 1:**
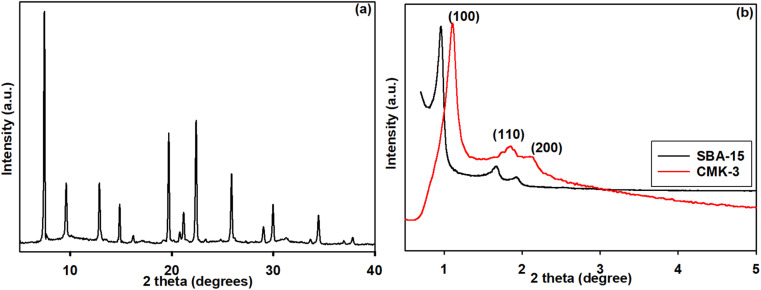
XRD patterns of the (a) hierarchical SAPO and (b) mesoporous silica SBA-15 and carbon CMK-3.

The XRD patterns displayed in Fig. 1a correspond to the hierarchical SAPO-5/34 samples. The peaks observed in the patterns can be attributed to different planes, such as (001), (101), (111), and (102) for CHA, and (100), (110), (200), (210), (002), (102), and (220) for AFI. The high degree of crystallinity evidenced by the XRD peaks suggests that the hierarchical structures with CHA/AFI framework have been successfully formed. The crystallite sizes of the hierarchical structures are also apparent from the XRD patterns, which agree well with the typical CHA/AFI-type structure.^35^ The synthetic method employed in this work enabled precise control of the crystallization process, leading to the formation of the hierarchical structure of SAPO-34 and SAPO-5 with CHA framework and AFI topology, respectively. The absence of diffraction peaks attributed to impurities in the XRD patterns indicates that the conditions for synthesizing the CHA-AFI-type SAPO zeolite, including the sources of Si and Al, and the template used, have been effectively optimized. The XRD results further reveal that the hierarchical structure with CHA framework and AFI topology is evident in the attainment of SAPO-5 and SAPO-34 planes, where preferential orientation growth is observed towards the typical (001) plane direction.^36^ These findings demonstrate the successful synthesis of the hierarchical SAPO-5/34 samples with high crystallinity and a well-defined CHA/AFI framework, using a controlled synthesis approach. Fig. 1b shows the typical small angle X-ray diffraction (XRD) pattern for the mesoporous silica template SBA-15 and the resulting carbon material CMK-3, obtained by removing the silica wall after carbonization. The SBA-15 low-angle XRD pattern, which is consistent with the hexagonal *P*6*mm* crystallographic space group with (100), (110), and (200) planes, demonstrates an outstanding structural order.^37^ Remarkably, the XRD pattern of CMK-3 exhibits a similar pattern to SBA-15, indicating that the carbon material dependably replicates the ordered structure of the template. Specifically, the appearance of diffraction peaks in the XRD pattern of CMK-3 that match the symmetry of SBA-15 provides strong evidence of structural transformation. Such an ordered arrangement of the carbon in CMK-3 could be elucidated by the obtained XRD patterns with planes (100), (110), and (200), similar to that of SBA-15.^38,39^ Therefore, it is obvious that the CMK-3 is an exact negative of the silicate SBA-15 template.

The high angle XRD patterns of SBA-15 and CMK-3 are shown in Fig. S1a.† The two broad diffraction peaks of (002) and (100) correspond to the graphitic structure. The *d*-spacing of the plane (002) was calculated to be 0.39 nm. The unit length of the hexagonal type CMK-3 ordered mesoporous carbon is found to be 10.8 nm. The XRD patterns of hierarchical SAPO-CMK-3 nanocomposite, APTES-functionalized SAPO-CMK-3 nanocomposite, and APTES-functionalized SAPO-CMK-3 crosslinked by GA, are shown in Fig. S1b–d.† In all the cases, (002) planes corresponding to the graphitic structure of CMK-3 are observed. This illustrates that the functionalization and cross linkage do not affect the structure of the hybrid HZ-CMK-3 supports.

**Fig. 2:**
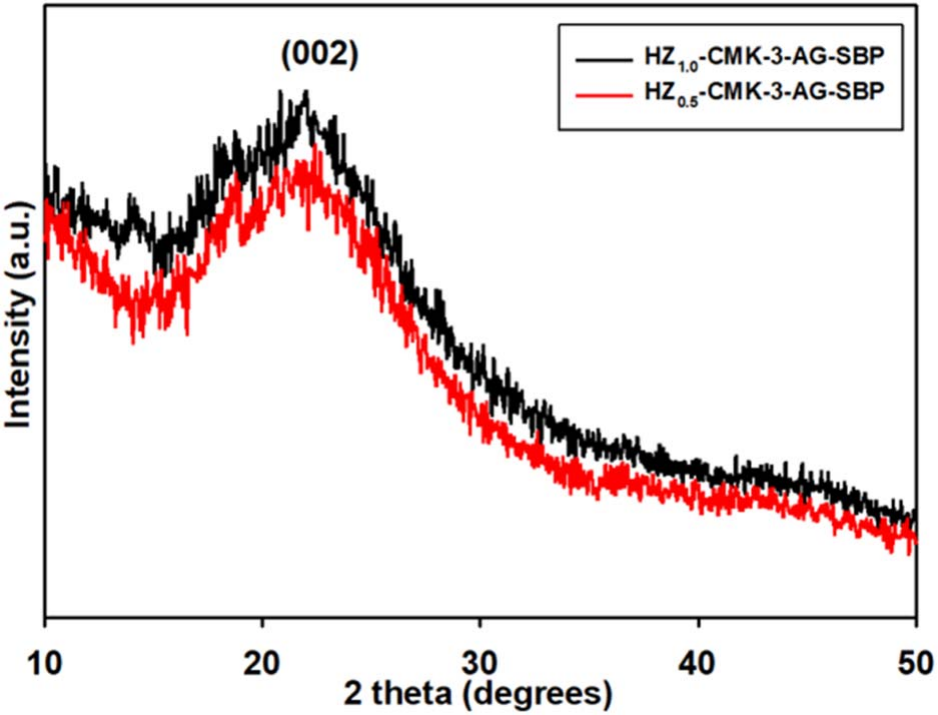
XRD pattern of SBP immobilized HZ nanocomposite.

The XRD pattern of SBP immobilized HZ nanocomposite is presented in Fig. 2. It was observed that the (002) plane is seen and it is obviously suggesting that the immobilization of SBP enzyme also did not affect the mesoporous carbon structure bonded to HZ within the functionalized hybrid system.

### 3.2. N_2_ sorption analysis

**Fig. 3:**
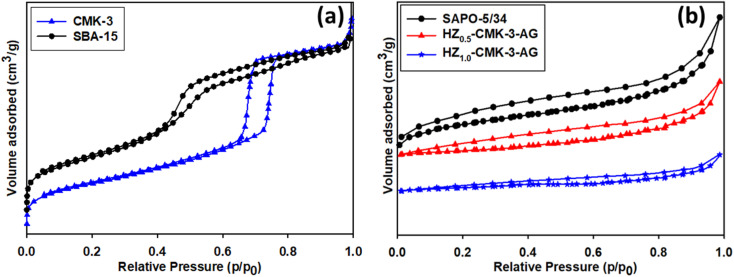
N_2_ adsorption–desorption isotherms of (a) SBA-15 and CMK-3; (b) hierarchical SAPO-5/34 and hierarchical SAPO/CMK-3-AG nanocomposites.

The textural properties of the prepared SBA-15 and CMK-3 materials were determined by N_2_ adsorption–desorption isotherms as shown in Fig. 3a. The mesoporous SBA-15 demonstrated type IV isotherm related to the mesopore region with a hexagonal pore system. The pore diameter and pore volume of SBA-15 were found to be 5.73 nm and 1.19 cm^3^ g^−1^. The uniform pore diameter of CMK-3 was 4.18 nm with a pore volume of 1.53 cm^3^ g^−1^ according to the isotherm results. The Brunauer–Emmett–Teller (BET) surface area is about 1315 m^2^ g^−1^ for CMK-3 and 735 m^2^ g^−1^ for SBA-15, respectively. N_2_ adsorption–desorption isotherms of hierarchical SAPO-5/34 and hierarchical SAPO/CMK-3-AG nanocomposites are shown in Fig. 3b. Their textural properties are presented in Table 1. The total pore volumes of the functionalized hierarchical composites were about 0.146 cm^3^ g^−1^ and 0.185 cm^3^ g^−1^. There is a decrease in the pore volume after the functionalization that ensures the modification occurred. Also, the effect of functionalization reflected in the BET surface area of the composites that tend to be decreased to 170.48 m^2^ g^−1^ and 86.63 m^2^ g^−1^ compared to the surface area of parent hierarchical SAPO-5/34 (324.43 m^2^ g^−1^).

**Table 1:** Textural properties of SAPO and nanocomposites obtained from N_2_ adsorption–desorption measurements

Sample	a *S*_BET_ [m^2^ g^−1^]	a *V*_total_ [cm^3^ g^−1^]	a *V*_micro_ [cm^3^ g^−1^]	a *V*_meso_ [cm^3^ g^−1^]	aAverage pore diameter [nm]
SAPO-5/34	324.43	0.209	0.176	0.034	1.870
HZ_0.5_-CMK-3-AG	170.48	0.146	0.039	0.107	2.162
HZ_1.0_-CMK-3-AG	86.63	0.185	0.040	0.145	1.643

a Calculated from the N_2_ adsorption isotherm data.

### 3.3. Morphology studies of SBA-15 and CMK-3

**Fig. 4:**
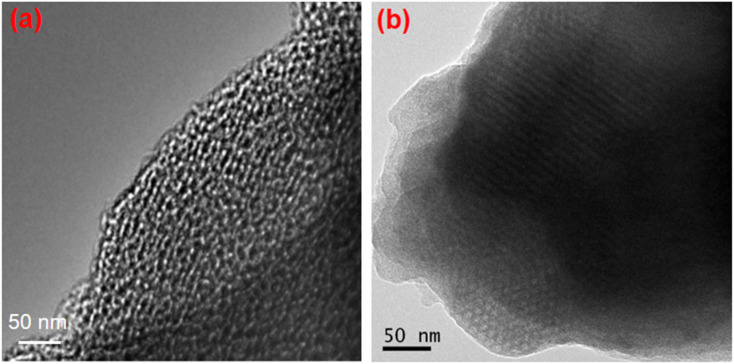
TEM images of (a) SBA-15 and (b) CMK-3.

The transmission electron microscopic (TEM) images of SBA-15 and CMK-3 are shown in Fig. 4. A hexagonal arrangement of silica with mesopores is clearly seen in the SBA-15 image. Moreover, the ordered mesoporosity in CMK-3 carbon is evident from the TEM analysis with a uniform pore size of about ∼4.2 nm. The mesoporous channels with uniform pores and regular mesopore arrangements consisting of carbon frameworks could be clearly observed. Hence, the CMK-3 is an inverse replica of the mesoporous SBA-15 template exhibiting long-range-ordered porous structure as confirmed by low angle XRD analysis.

### 3.4. FTIR spectroscopy

**Fig. 5:**
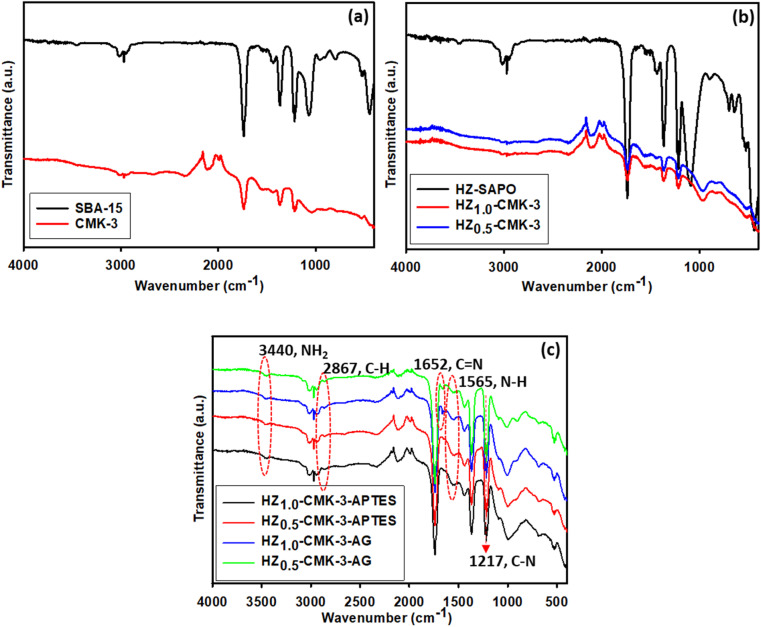
FTIR spectra of (a) SBA-15 and CMK-3, (b) HZ-SAPO/CMK-3, (c) HZ-CMK-3-APTES and HZ-CMK-3-AG *i.e.*, after APTES functionalization and crosslinking with GA.

The functional groups in the hybrid structures of the samples were determined by FTIR spectral analysis. The FTIR spectra of SBA-15 and CMK-3 are shown in Fig. 5a. The IR bands of SBA-15 correspond to those reported for surface silanols and adsorbed H_2_O molecules observed at 3423 cm^−1^, and the related deformational vibrations appeared around 1632 cm^−1^. The absorption bands observed for SBA-15 at 1082, 804, and 461 cm^−1^ are assigned to asymmetric, symmetric stretching and bending of Si–O–Si groups of the mesoporous materials.^40,41^ The IR spectra of CMK-3 demonstrate a characteristic band at around 3410 cm^−1^ ascribed to –OH stretching and the –COOH groups bending vibration at 1589 cm^−1^. Also, C=C and C=O stretching vibrations were observed at 2371 cm^−1^, 1733 cm^−1^, and 1581 cm^−1^ and C–C–O bond at 1120 cm^−1^.

The FTIR spectra of HZ-SAPO and HZ-CMK-3 are presented in Fig. 5b, where the functional groups of related characteristics in hybrids were elucidated. The IR bands in HZ-SAPO spectra revealed the tetrahedral SiO_4_ corresponding to T–O bending, and T–O bending in D-6 rings, those were identified at 493 cm^−1^, and 624 cm^−1^, respectively. CHA framework in the hierarchical SAPO material is confirmed by D-6 rings functionalities that endorse the hierarchical structure.^42^ The IR bands at 702 cm^−1^ and 1081 cm^−1^ assigned to symmetric stretching vibration (Al–O and/or P–O) and asymmetric stretching vibration (O–P–O) were confirmed. Besides, Si–OH–Al groups with bridging OH groups in the hybrid structure were observed at 3263 cm^−1^_._ For HZ_1.0_-CMK-3 and HZ_0.5_-CMK-3 samples, a similar kind of peaks representing hierarchical zeolite structure (T–O bending in D-6 rings, and Al–O, P–O, O–P–O stretching) and CMK-3 functional elements (COOH, C=C, and OH) was observed with less intensity compared to HZ-SAPO due to the combination of HZ and CMK-3 functional groups as overlapping observance. Fig. 5c shows the IR spectra of functionalized HZ-CMK-3 by APTES and cross linked with GA, respectively. The peak at 2867 cm^−1^ in APTES functionalized samples corresponds to the stretching vibration of the C–H bond of the propyl group present in APTES.^43^ The IR band at 3440 cm^−1^ corresponds to NH stretching in NH_2_, the peak observed at 1565 cm^−1^ is ascribed to N–H bending and moreover, a peak at 1217 cm^−1^ is ascribed to C–N stretching vibration in amine those arising from the APTES functionalization. All of these observations on C–H, and N–H groups confirm the functionalization of APTES in the hybrid functionalized HZ-CMK-3-APTES for HZ_1.0_-CMK-3 and HZ_0.5_-CMK-3 ratios. After the addition of GA, a new peak emerged at 1652 cm^−1^ corresponding to C=N stretching that arose from the aldehyde and amine group bonding. Therefore, the functionalized and crosslinked (APTES and GA) hybrid HZ-CMK-3-AG is well confirmed with the necessary functionalities compared to non-functionalized materials.

**Fig. 6:**
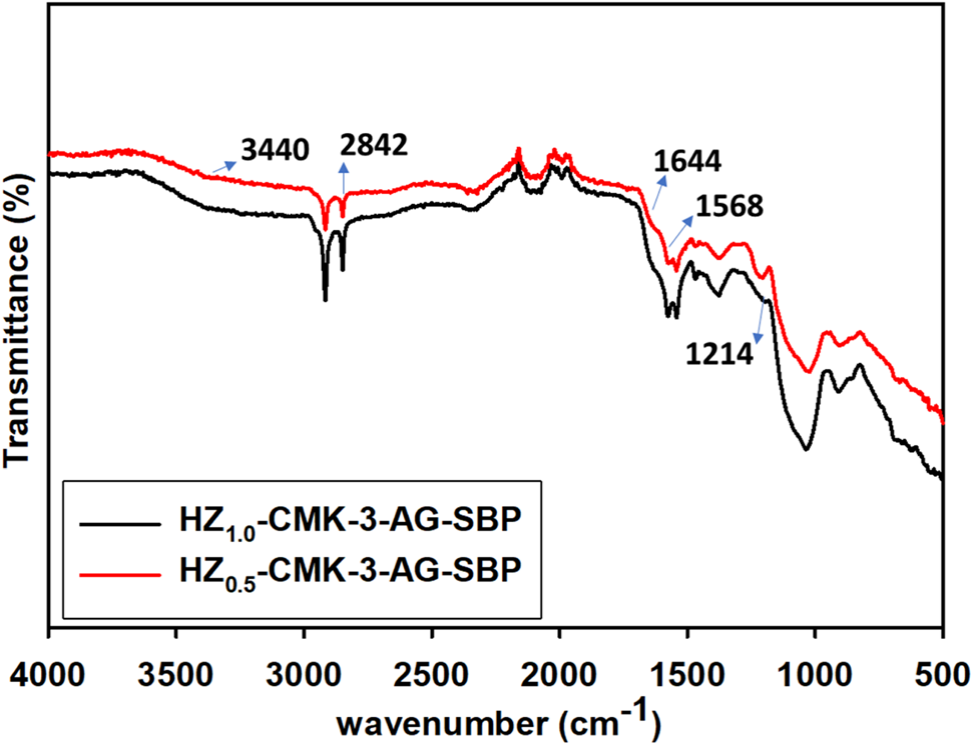
FTIR spectra of SBP immobilized HZ nanocomposite.

The FTIR spectra of SBP immobilized HZ_1.0_-CMK-3-AG-SBP and HZ_0.5_-CMK-3-AG-SBP nanocomposite is shown in Fig. 6. From the observation, reveals the characteristic band of NH stretching at 3440 cm^−1^, CH groups at 2842 cm^−1^, C=N at 1644 cm^−1^, NH bending 1568 cm^−1^ and C–N stretching 1214 cm^−1^. C–H groups in SBP also appeared at the same region that was observed for the APTES propyl group in the functionalized materials and with more intensity in the SBP immobilized samples. Moreover, the peak at 1644 cm^−1^ that corresponded to C=N bond occurred from the bonding of the –NH_2_ group of APTES and –CHO group of GA was observed to possess a wide region due to the SBP immobilized enzyme. Furthermore, C=N stretching was observed at 1652 cm^−1^ for APTES functionalized materials that now fell in the lower vibration region at 1644 cm^−1^ for SBP immobilized materials, respectively. The obtained results indicated the modifications of such functionalities occurred from the covalent bonding of SBP enzyme resulting in efficient immobilization. Correspondingly, N–H, C–N, and distinct C=N functional groups are observed in the spectra of immobilized samples confirming the functionalization of APTES, and GA along with the immobilization of SBP enzyme onto the surface as observed for HZ_1.0_-CMK-3-AG-SBP and HZ_0.5_-CMK-3-AG-SBP.

### 3.5. Raman spectroscopy

**Fig. 7:**
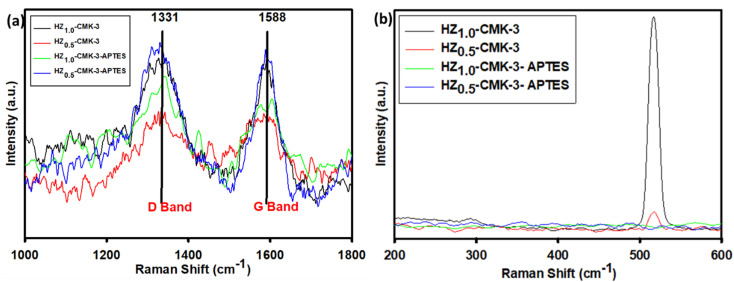
Raman spectra of hierarchical SAPO/CMK-3 and hierarchical SAPO/CMK-3-APTES functionalized (a) higher shift, and (b) lower shift region.

The Raman spectra of a hybrid HZ/CMK-3 mesoporous carbon nanocomposite is shown in Fig. 7. The spectra demonstrate that the hybrid nanocomposite exhibits a prominent Raman band at 1588 cm^−1^, referred to as the G band. This band forms in a two-dimensional hexagonal lattice of graphene sheets due to the in-plane vibration of carbon atoms that are sp^2^-associated (referred to as the E_2g_ mode).^44^ This signifies the presence of a well-organized structure within the nanocomposite. Additionally, another peak is observed at 1331 cm^−1^, stated as the D band.^44,45^

The D band indicates the existence of defects or partially disordered (amorphous) structures within the nanocomposite. Its presence suggests that some imperfections or irregularities are present in the arrangement of carbon atoms. The simultaneous appearance of the G band and D band confirms the effective assembly of CMK-3 ordered mesoporous carbon with HZ in both the HZ@CMK-3 and HZ@CMK-3-APTES composites, as illustrated in Fig. 7a. This demonstrates the successful integration of HZ and CMK-3, resulting in a nanocomposite with a well-structured framework while also incorporating some amorphous regions or defects. The lower shift region in Raman spectra revealed the characteristic band of amorphous silica at 516 cm^−1^ (Fig. 7b).

### 3.6. Morphology studies of parent materials, functionalized and cross-linked hierarchical composites

**Fig. 8:**
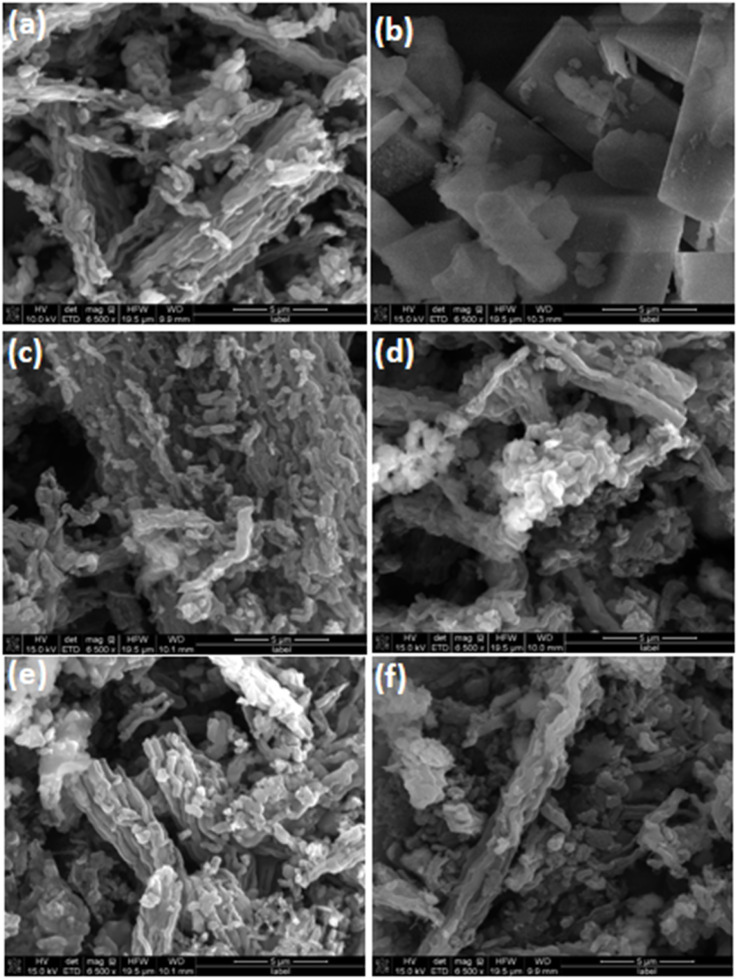
SEM images of (a) CMK-3; (b) HZ; (c) HZ-CMK-3-(0.5); (d) HZ-CMK-3-(1.0); (e) HZ-CMK-3-APTES (0.5); (f) HZ-CMK-3-APTES (1.0). Scale bar: 5 μm.

**Fig. 9:**
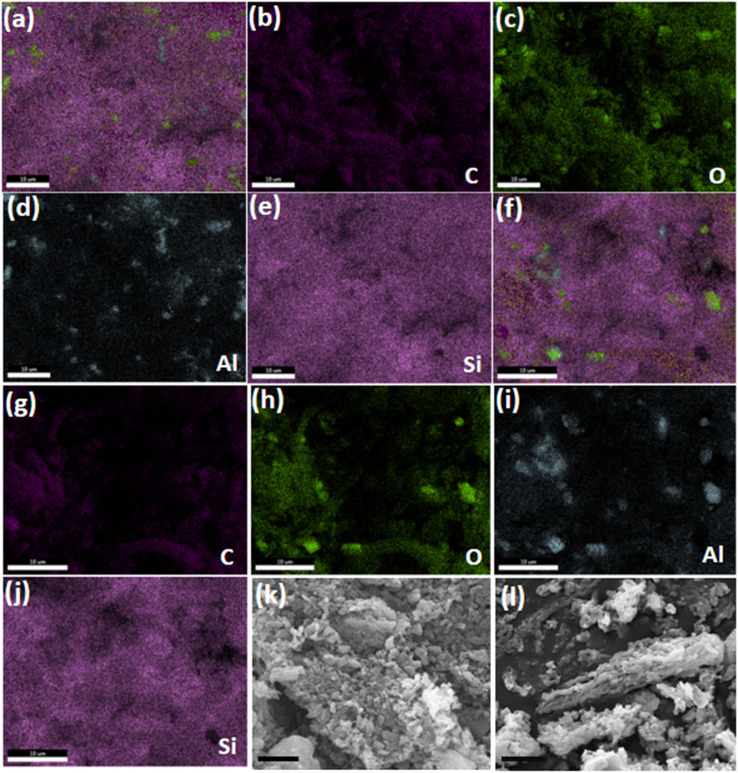
EDS mapping of HZ-CMK-3-APTES (0.5) (a–e), HZ-CMK-3-APTES (1.0) (f–j); scale bar: 10 μm, (k) HZ_0.5_-CMK-3-AG and (l) HZ_1.0_-CMK-3-AG; scale bar 5 μm.

The SEM images of CMK-3, HZ, HZ-CMK-3, and APTES functionalized HZ-CMK-3 are presented in Fig. 8. In Fig. 8a, it was observed that the CMK-3 particles in their original state exhibit a clean and organized worm-like structure without any additional features on the surface. Even after undergoing the carbonization process, CMK-3 maintains its ordered mesoporous structure without experiencing any significant damage. Fig. 8b shows the zeolitic structure with the combination of cubic and flat-like morphology representing a hierarchical SAPO family of materials. In Fig. 8c and d, a composite structure consisting of HZ and CMK-3 rod-bundle-like constructions were observed forming a successful hybrid element for HZ_0.5_-CMK-3 and HZ_1.0_-CMK-3, respectively. After APTES functionalization, the materials remained with a similar morphology as observed for HZ_0.5_-CMK-3-APTES and HZ_1.0_-CMK-3-APTES. The respective functionalization and structural order with microscopic, FTIR, and XRD evaluation, suggests that the functionalization is effective and stable. Fig. 9a–j shows the clear EDS mapping of the hierarchical nanocomposite materials. The mapping images exhibit the distribution of the elements (C, Al, Si, O), suggesting a close proximity of these elements on the surface of HZ-CMK-3 nanocomposites.

**Fig. 10:**
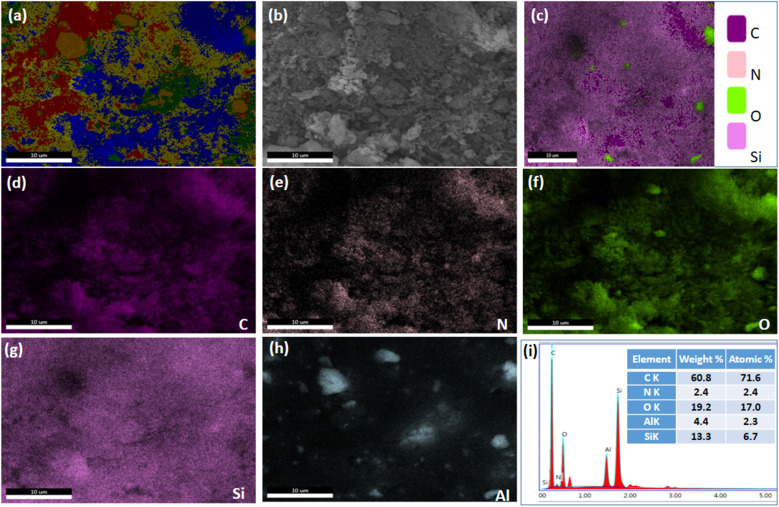
(a–h) SEM images (scale bar: 10 μm) and (i) EDAX spectra of HZ_0.5_-CMK-3-AG.

**Fig. 11:**
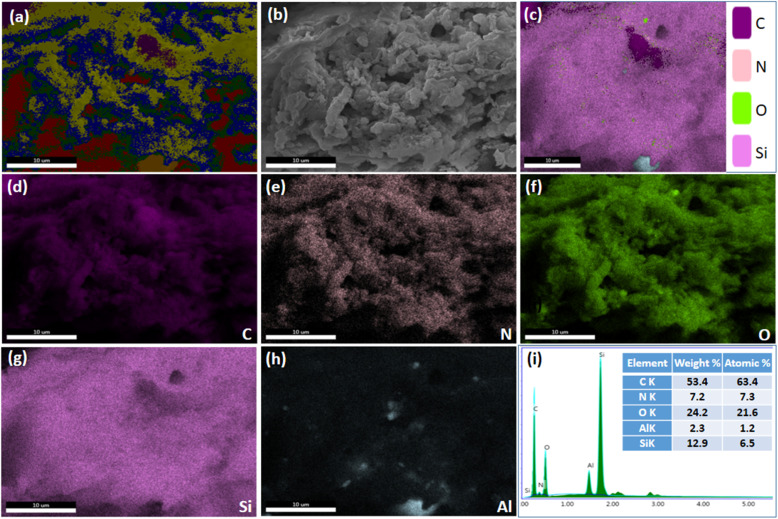
(a–h) SEM images (scale bar: 10 μm) and (i) EDAX spectra of HZ_1.0_-CMK-3-AG.

**Fig. 12:**
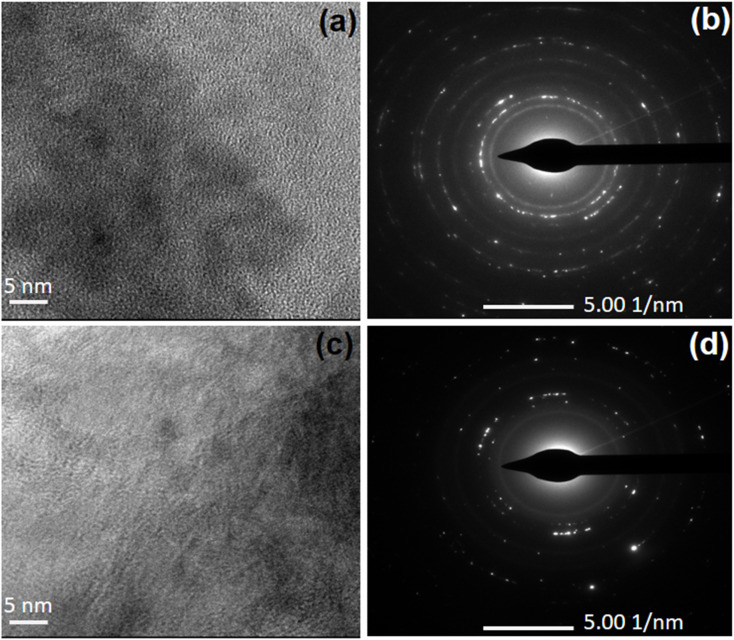
TEM and SAED images of (a and b) HZ_0.5_-CMK-3-AG and (c and d) HZ_1.0_-CMK-3-AG.

SEM images of HZ_0.5_-CMK-3-AG and HZ_1.0_-CMK-3-AG are presented in Fig. 9k and (l). The overall mapping of HZ_0.5_-CMK-3-AG and HZ_1.0_-CMK-3-AG are shown in Fig. 10 and 11, respectively. After the crosslinking of GA, the elements such as C, N, O, Si and Al was well observed for all the samples. This confirms the successful amine functionalization within the composites even after the GA treatment. The corresponding weight and atomic percentages are provided in the inset of EDAX profile. Additionally, the morphology of the GA crosslinked materials was observed by TEM images with respective selected area electron diffraction (SAED) patterns as shown in Fig. 12. The SAED patterns are assigned to the characteristics of single crystals with multiple nanodomains associated with SAPO zeolite materials.^46^ Such crystallites are aligned along the same crystallographic axis, and observed to be fused together due to the grain boundaries that are attributed to the hierarchical building blocks.^47^

### 3.7. Immobilization of SBP activity studies

**Fig. 13:**
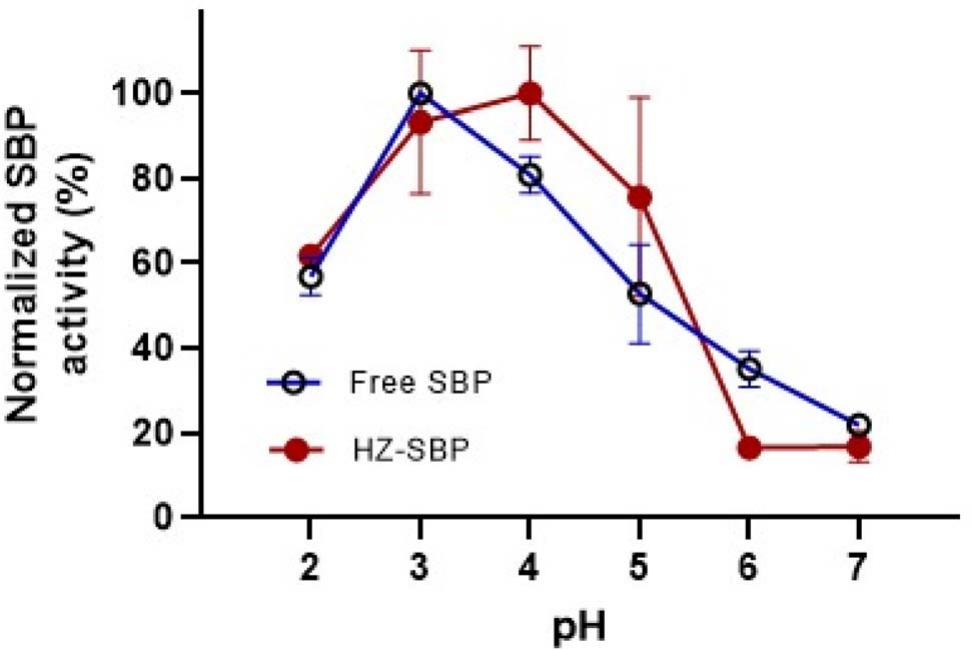
Effect of pH on SBP enzyme activity.

The effect of pH on free SBP activity is considerably important where the immobilized SBP onto HZ-CMK-3-AG could be applied in various biocatalytic and adsorbent applications under pH regulated conditions. The effect of pH on SBP activity was investigated at room temperature and shown in Fig. 13. As shown in the figure, free SBP exhibited highest activity at pH 3. On increasing the pH level, the enzyme activity gradually decreased to zero activity at pH 7.0 and 8.0.

Similarly, the pH profile of immobilized SBP was evaluated and found to follow the same trend as its free counterpart, with peak activity in the acidic range (pH 3–5). From this observation, it could be suggested that SBP can be employed as efficient biocatalysts and adsorbents involving the reactants solubility and reactivity at lower pH conditions for specific applications.

**Fig. 14:**
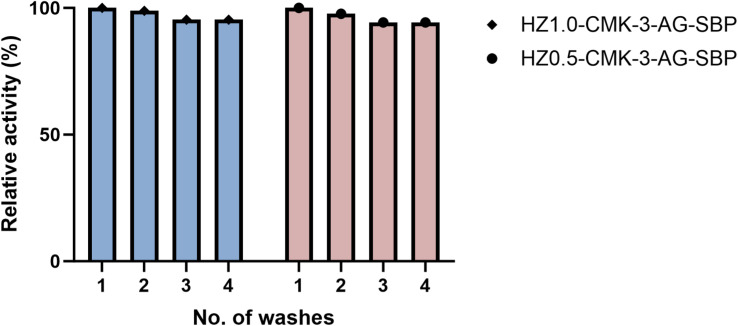
SBP activity for four washing.

Reusability, as well as leaching of the components, are imperative and vital properties in the biocatalytic processes to perform with equivalent utility recurrently toward real-time applications. For the cycle test, 10 mg slurry of HZ_*x*_-CMK-3-AG-SBP was mixed with 1 mL of distilled water and then shaken gently for 2 min, and then spun for 5 min to separate the supernatant for testing the activity. To this decant solution was mixed with a “master mix” for the analysis. This was washing number 1 with the slurry test. Then, the same slurry was used further with 1 mL of water, and the same procedure was applied until four cycles, and the results are shown in Fig. 14. SBP activity was investigated to assess the repeated usability of immobilized SBP with successive cycles. The activity was found to be 94.3% after 3 washes and tend to maintain the activity at 4 wash numbers. There was no active enzyme leaching from the support as observed, under the reaction conditions for consecutive recycles. This suggested that the SBP enzyme had strong adherence with strong covalent-co-ordinate bonding onto the functionalized HZ_*x*_-CMK-3-AG nanocomposites.

## 4. Conclusions

In summary, the controlled fabrication of hierarchical SAPOs with the CHA framework and AFI topology was successfully carried out. The obtained HZ-SAPO was further reacted with CMK-3 (prepared from the SBA-15 template) to obtain a hybrid HZ-CMK-3. Various physicochemical characterization XRD, N_2_ isotherms, FTIR spectra, Raman spectra, SEM, and TEM evidenced the structure and surface morphology of the as-synthesized materials. These HZ-CMK-3 hybrids were functionalized by employing APTES and crosslinked with GA. The successful functionalization and crosslinking were confirmed through spectroscopic analysis, demonstrating the effective incorporation of key functional groups. The resulting hybrid HZ_*x*_-CMK-3-AG materials were used for the immobilization of SBP enzyme. The corresponding functionalities before and after immobilization were evidenced by the XRD and FTIR results. The controlled synthesis approach of hierarchical zeolites and CMK-3 followed by effective functionalization, crosslinking, and successful immobilization has led to the development a novel inorganic-bioorganic hybrid nanocomposites. Further, the activity of SBP immobilized HZ_*x*_-CMK-3-AG was determined by standard methods. The activity results revealed the applicability of biocatalysts under lower pH (4.0) conditions. Furthermore, the effective SBP immobilization was well evident from the recyclable nature and strong bonding within the fabricated bio nanocomposite HZ-CMK-3-AG-SBP. The findings of this work highlight the successful development of a novel HZ-CMK-3-AG-SBP bio-nanocomposite with enhanced enzyme stability, recyclability, and activity under acidic conditions, demonstrating its potential for application in industrial biocatalytic processes. This approach provides a valuable framework for designing efficient and sustainable biocatalysts for diverse environmental and chemical transformations.

## Data availability

All data generated or analyzed during this study are included in this manuscript.

## Author contributions

Prabhu Azhagapillai: conceptualization, data curation, formal analysis, methodology, writing – original draft. Karthikeyan Gopalsamy: data curation, formal analysis, methodology, writing – original draft, reviewing and editing. Israa Othman: formal analysis. Nada I. Alhatti: formal analysis. Mohammad Abu Haija: supervision, reviewing and editing. Syed Salman Ashraf: investigation, resources, supervision, visualization, funding acquisition, reviewing and Editing.

## Conflicts of interest

The authors declare that they have no known competing financial interests or personal relationships that could have appeared to influence the work reported in this paper.

## Supplementary Material

RA-015-D4RA07503J-s001
