# Supplementary material for: Immobilization of soybean peroxidase enzyme on hierarchical zeolite-ordered mesoporous carbon nanocomposite and its activity
Source: RSC Adv. 2025 Feb 20;15(8):5781–94. doi: 10.1039/d4ra07503j (PMC11840808; doi:10.1039/d4ra07503j)
Supplement: RA-015-D4RA07503J-s001 [file RA-015-D4RA07503J-s001.pdf]

Supplementary Information (SI) for RSC Advances.
This journal is © The Royal Society of Chemistry 2025

Supporting Information

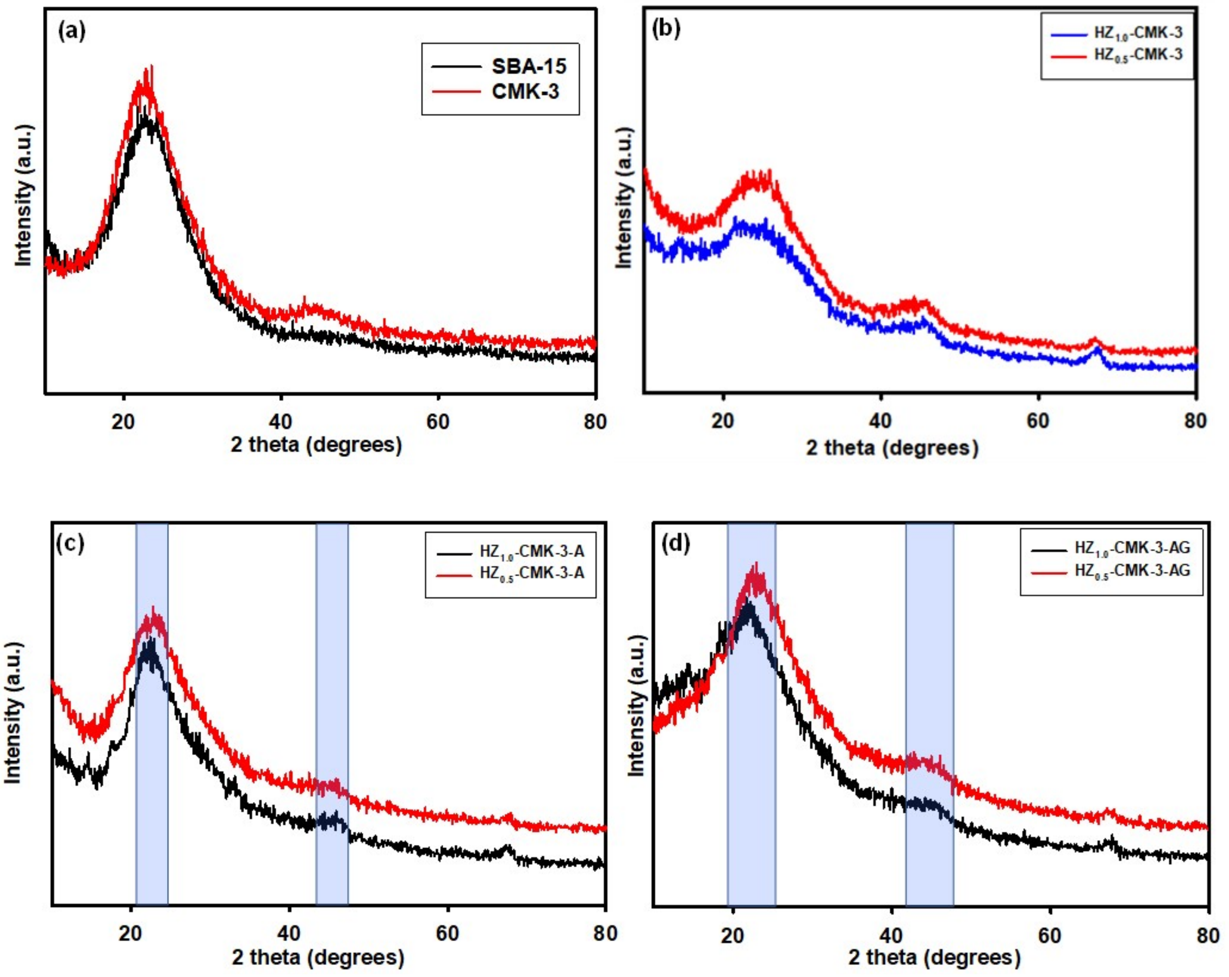

Fig. S1. High angle XRD patterns of (a) SBA-15, CMK-3, (b) nanocomposite of hierarchical SAPO/CMK-3, (c) After APTES modification, (d) After Crosslinking with GA.
